# Metatranscriptomic analysis of the gut virome in Muscovy ducks reveals a novel duck reovirus potentially associated with hepatic and splenic hemorrhage

**DOI:** 10.3389/fimmu.2025.1680275

**Published:** 2025-10-23

**Authors:** Chuang Liu, Qixiang Kang, Xuanci Wang, Fangui Zeng, Yihui Huang, Yiqin Yang, Rui Geng, Jiamin Liao, Xin Luo, Zhuanqiang Yan, Lijuan Yin, Sheng Cao, Feng Chen, Hao Zhang, Ouyang Peng, Yongchang Cao

**Affiliations:** ^1^ State Key Laboratory of Biocontrol, School of Life Sciences, Sun Yat-sen University, Guangzhou, China; ^2^ Wens Foodstuffs Group Co., Ltd., Yunfu, China; ^3^ College of Animal Science, South China Agricultural University, Guangzhou, China

**Keywords:** Muscovy ducks, metatranscriptomic sequencing, intestinal viral communities, novel duck reovirus, hepatic and splenic hemorrhage

## Abstract

**Introduction:**

Ducks rank among the most important sources of animal protein globally, yet hepatic and splenic hemorrhage and necrosis in Muscovy ducks present a critical challenge to the poultry industry. The causes behind such diseases are often multifaceted, involving both established and newly emerging pathogens.

**Methods:**

In this study, we leveraged metatranscriptomic sequencing to profile the intestinal viral communities of healthy and diseased Muscovy ducks from a Guangdong Province farm that experienced a hepatic and splenic hemorrhage in June 2024.

**Results:**

Our findings revealed marked differences in viral community profiles between the two groups, with the diseased cohort exhibiting higher α-diversity. Taxonomic analyses across multiple levels uncovered significant variations in viral composition, including shifts in phylums like Uroviricota and families such as Demerecviridae. At the genus and species levels, several bacteriophages and eukaryotic viruses displayed differential abundance. Notably, Avian orthoreovirus was detected exclusively in diseased ducks, with a specific novel duck reovirus (NDRV) validated via RT-qPCR as a potential contributor to hepatic and splenic pathogenesis. In contrast, known pathogens such as Duck hepatitis A virus (DHAV) and Fowl adenovirus serotype 4 (FAdV-4) were not detected.

**Discussion:**

This study constitutes the first comprehensive analysis of the Muscovy duck gut virome, highlighting NDRV as a potential causative agent and emphasizing the utility of metatranscriptomics in pathogen discovery.

## 1 Introduction

Ducks represent an important source of animal protein and are widely consumed around the world, especially in Asia (1). However, hemorrhage and necrosis of the liver and spleen, particularly in Muscovy ducks (*Cairina moschata*), pose a serious threat to the poultry industry (2). These pathological manifestations could be caused by a variety of pathogens, and novel causative agents keep emerging over time. Previous studies have identified Duck Hepatitis A Virus (DHAV) as one of the primary etiological agents responsible for hepatic hemorrhage in ducks, leading to liver necrosis and hemorrhagic lesions that severely impact animal health and farming profitability (3, 4). In addition to DHAV, fowl adenovirus serotype 4 (FAdV-4) has also been recognized as a significant pathogen causing hepatic hemorrhage in ducks (5, 6). More recently, novel duck reovirus (NDRV) has been identified as another causative agent. NDRV infection has also been associated with severe splenic necrosis, which might result in immunosuppression and secondary infections (7, 8). Moreover, NDRV can be transmitted vertically, further exacerbating its potential for widespread dissemination within duck populations (9). Collectively, these findings underscore the complex etiology of hepatic and splenic hemorrhagic disease in ducks, which often involves co-infection or emergence of novel pathogens. Such infections not only compromise duck health but also lead to substantial economic losses in poultry production. Therefore, early detection and accurate diagnosis of both known and emerging pathogens are critical for the development of effective prevention and control strategies.

Viral metagenomics is an emerging and powerful tool that has grown increasingly valuable for the identification of novel pathogens (10). By enabling the comprehensive characterization of complex microbial communities without prior knowledge of the target organisms, metaranscriptomic or metagenomic approaches and newly developed deep-learning based algorithms allow the unbiased detection of both known and previously unrecognized viruses (11–14). This technology has already demonstrated great promise in the fields of public health surveillance and infectious disease diagnostics (15). In contrast, the application of viral metagenomics in poultry remains limited. Nevertheless, several studies have shown its utility in avian virus monitoring. For instance, metagenomic analysis of samples from six poultry farms, including chicken, duck, and goose farms, in eastern China enabled the simultaneous detection of multiple avian viruses, including avian influenza virus and Newcastle disease virus (16). Similarly, metatranscriptome sequencing has been employed to profile the diversity and abundance of avian viruses in live poultry markets in Guangdong Province, further demonstrating its diagnostic and epidemiological potential (17). In addition to known pathogens, this approach has proven invaluable in the discovery of novel viruses from environmental samples that would otherwise evade detection by conventional techniques (18). It has also been successfully applied to wildlife studies for virus identification and characterization (19). Despite the growing interest and impact in viral metagenomics, its application in domestic poultry—particularly in ducks—remains underexplored.

In June 2024, a Muscovy duck farm in Guangdong Province reported severe cases of hepatic and splenic hemorrhage and necrosis, the etiology of which remained unknown. To date, no studies have characterized the intestinal virome of Muscovy ducks. To address this gap and investigate potential causative pathogens, we applied metatranscriptomic sequencing to characterize and compare the intestinal viral communities of diseased and healthy ducks. Our comparative analysis found that *Avian orthoreovirus* was significantly enriched in the intestines of diseased individuals, suggesting its potential role as a causative agent. This finding was further supported by RT-qPCR validation, which confirmed the presence of novel duck reovirus (NDRV) in affected ducks. This study represents the first comprehensive analysis of the intestinal virome in Muscovy ducks and provides critical foundational data for future research into duck gut health. Furthermore, our identification of NDRV as a potential etiological agent demonstrates the practical utility of metatranscriptomics in pathogen discovery, offering a useful model for rapid diagnosis and detection of unknown pathogens in poultry farming.

## 2 Materials and methods

### 2.1 Ethics statement

The experimental protocol was reviewed and approved by the Institutional Animal Care and Use Committee (IACUC), Sun Yat-Sen University, Guangzhou, China (Approval ID: SYXK-2023-0112). All procedures involving animals were conducted in line with institutional guidelines for animal welfare and ethical research practices.

### 2.2 Sample collection

In June 2024, a severe outbreak characterized by marked hepatic and splenic hemorrhage occurred in 1–2-week-old Muscovy ducks (*Cairina moschata*) at a commercial farm in Guangdong Province, China. Fresh cecal contents were collected from diseased ducks immediately after necropsy. For sample pooling, contents from every three diseased ducks were combined into one biological replicate, yielding 10 pooled samples for the diseased group. Age-matched healthy Muscovy ducks from the same farm served as controls, with cecal contents collected using the same pooling strategy. All samples were immediately flash-frozen in liquid nitrogen immediately after collection, transported to the laboratory under cryogenic conditions, and stored at -80 °C until subsequent processing.

### 2.3 Total RNA extraction for sequencing

Total RNA was extracted from cecal content samples using the RNAPowerSoil Total RNA Isolation Kit (MoBio Laboratories, Carlsbad, CA, USA; Cat. No. 12866-25) according to the manufacturer’s protocol. RNA concentration and purity were then assessed using a Qubit Fluorometer and a Nanodrop 2000 UV spectrophotometer. Only samples meeting the following criteria were used for library construction: minimum concentration ≥ 80 ng/μL, A260/A280 ratio ≥ 1.8, and total RNA amount ≥ 2 μg per sample.

### 2.4 Viral metatranscriptomic library construction

Ribosomal RNA (rRNA) was depleted by hybridizing rRNA-specific biotinylated probes to total RNA, followed by capturing of rRNA-probe complexes using streptavidin-coated magnetic beads (Illumina, San Diego, CA, USA). The remaining mRNA was purified via ethanol precipitation. cDNA library construction was carried out using the TruSeq Stranded mRNA LT Sample Prep Kit (Illumina) according to the manufacturer’s instructions, including reverse transcription, adaptor ligation, and PCR amplification. Library integrity was validated using an Agilent Bioanalyzer 2100 with the Agilent High Sensitivity DNA Kit (Agilent Technologies, Santa Clara, CA, USA), with qualification requiring a single distinct peak without adapter dimers. Library quantification was performed using the Quant-iT PicoGreen dsDNA Assay Kit (Thermo Fisher Scientific) on a Promega QuantiFluor fluorometer, with only libraries achieving a concentration ≥ 2 nM deemed qualified for sequencing.

### 2.5 Metatranscriptomic sequencing

Individual qualified libraries, each bearing unique indices, were diluted to 2 nM and pooled in equimolar ratios based on target sequencing depth. Pooled libraries were denatured into single-stranded DNA using 0.2 N NaOH and sequenced on an Illumina NovaSeq 6000 platform, generating 2 × 150 bp paired-end reads.

### 2.6 Metatranscriptomic data analysis

Raw sequencing data were processed using the Hecatomb pipeline (20), a specialized, high-performance tool optimized for virome analysis of metatranscriptomic datasets. The workflow included the following steps: (1) removal of adapter sequences, primers, and low-quality reads using Trimmomatic (21); (2) depletion of host genomic and transcriptomic sequences via alignment to the Muscovy duck reference genome (GenBank: GCA_048319975.1) utilizing Bowtie2 (22); (3) reduction of sequence redundancy via clustering with CD-HIT-EST (23); (4) generation of sequence count tables. Taxonomic classification was performed using an iterative search strategy implemented in MMSeqs2 (24). Initially, processed sequences were aligned to a virus-specific protein database (RefSeq Viral Proteins) using a translated nucleotide-to-protein (tblastx) approach. Sequences assigned to a viral taxonomy were subjected to secondary validation against a comprehensive reference database containing sequences from bacteria, plants, vertebrates, fungi, and other domains to eliminate false positives. This two-step strategy balanced computational efficiency with classification accuracy, avoiding the prohibitive cost of direct searches against large general databases.

### 2.7 Real-time quantitative PCR validation

For RT-qPCR validation, 2 g aliquots of cecal samples were homogenized in 500 μL ice-cold phosphate-buffered saline (PBS) by vortexing vigorously. After centrifugation at 12,000 × g for 10 min at 4°C, the supernatant was collected for viral nucleic acid extraction. Total viral DNA/RNA was isolated using the Vazyme FastPure Viral DNA/RNA Mini Kit V2 (Vazyme Biotech, Nanjing, China; Cat. No. RC313) following the manufacturer’s protocol, with all procedures performed on ice to maintain nucleic acid integrity. RNA concentration and purity were determined spectrophotometrically (NanoDrop 2000; Thermo Fisher Scientific, Wilmington, DE, USA), with samples exhibiting A260/A280 ratios between 1.8 and 2.0 considered suitable for downstream analysis. Reverse transcription was performed using the PrimeScript™ RT Reagent Kit with gDNA Eraser (Takara Bio, Shiga, Japan) according to the manufacturer’s instructions, incorporating a genomic DNA elimination step prior to cDNA synthesis. Quantitative PCR amplification was conducted using SYBR^®^ Premix Ex Taq II (Tli RNaseH Plus; Takara Bio) on a LightCycler 480 II Real-Time PCR System (Roche Diagnostics, Basel, Switzerland). The thermal cycling protocol consisted of an initial incubation at 50°C for 2 min, followed by denaturation at 95°C for 10 min, and 40 cycles of 95°C for 15 s, 60°C for 15 s, and 72°C for 30 s. All reactions were performed in triplicate, with appropriate negative controls included in each run. Primer sequences targeting specific viral genes are provided in **Supplementary Table S1** (25–27).

### 2.8 Statistical analysis and visualization

Partial least squares discriminant analysis (PLS-DA) was performed using the package mixOmics (28) to visualize differences in viral community composition between groups. Heatmaps of viral abundance were generated using the ComplexHeatmap (29) package, and scatter plots were constructed with ggplot2. Statistical comparisons of viral abundance between healthy and diseased groups were conducted using Student’s t-test, with p-values < 0.05 considered statistically significant. All analyses were performed in R version 4.2.1.

## 3 Results

### 3.1 Increased viral diversity characterizes the gut virome of diseased ducklings

**Figure 1:**
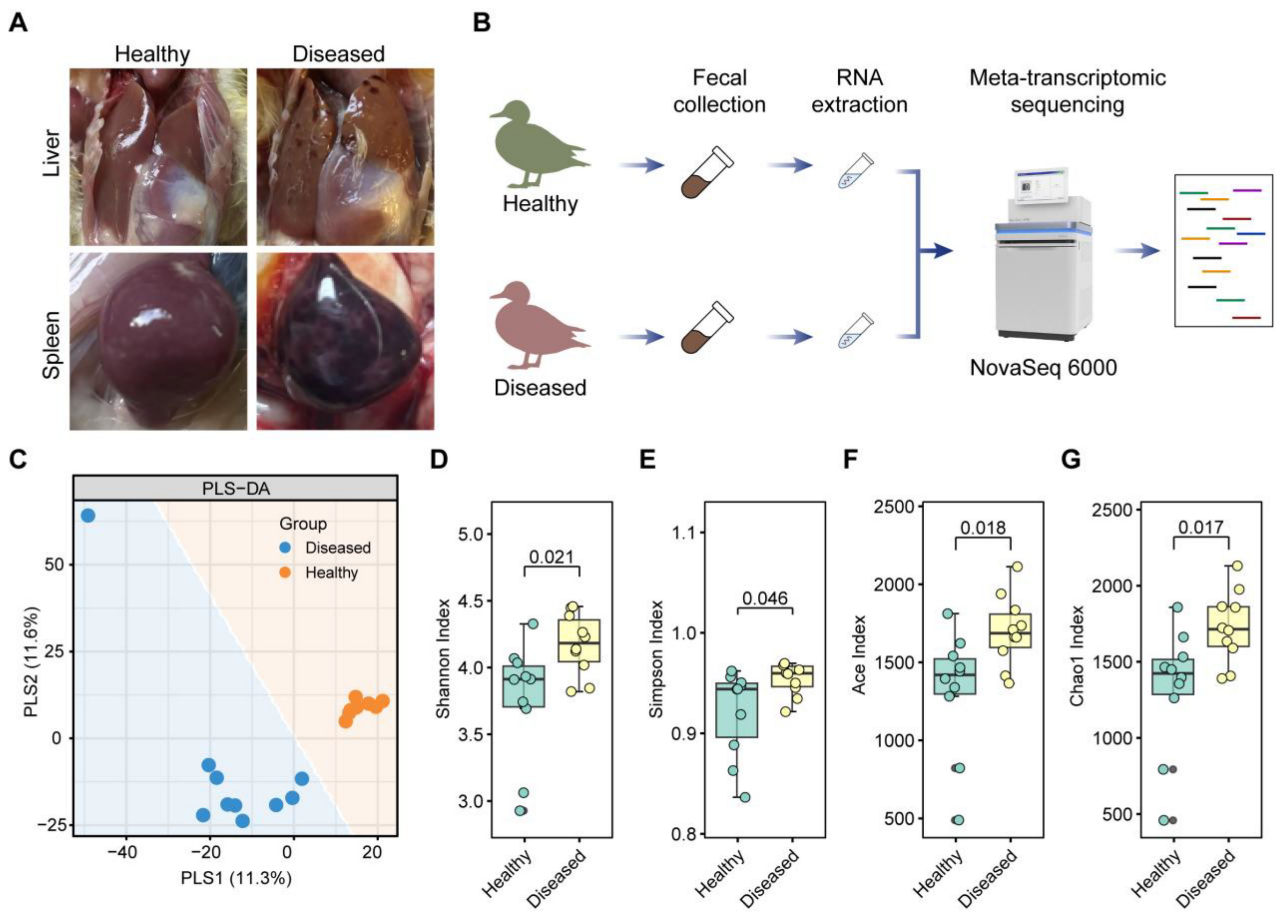
Overview of clinical sample collection and intestinal metatranscriptomic sequencing. **(A)** Representative gross pathological features of both healthy and diseased Muscovy ducks; **(B)** Schematic workflow illustrating the steps involved in intestinal metatranscriptomic sequencing; **(C)** Partial least squares discriminant analysis (PLS-DA) of virome profiles; **(D–G)** Alpha diversity indices of the duck gut virome, including Shannon **(D)**, Simpson **(E)**, ACE **(F)**, and Chao1 **(G)**.

In June 2024, a sudden surge in mortality occurred at a Muscovy duck farm in Guangdong Province, triggering an urgent pathological investigation. Necropsies revealed extensive punctate hepatic hemorrhages and diffuse splenic bleeding (**Figure 1A**), raising suspicion of a viral etiology. However, the exact causative virus remained undetermined. To identify potential pathogens, we extracted cecal contents from both symptomatic and asymptomatic ducks and conducted metatranscriptomic sequencing. Following the removal of host and bacterial sequences, the remaining reads were annotated using viral databases (**Figure 1B**). Partial least squares discriminant analysis (PLS-DA) revealed clear clustering that separated diseased from healthy samples, reflecting strong within-group similarity and substantial inter-group differences in viral composition (**Figure 1C**). Moreover, alpha-diversity indices were significantly elevated in diseased ducks, suggesting greater viral richness and complexity (**Figures 1D–G**). These findings provide a comparative dataset of the gut virome in healthy versus diseased Muscovy ducks, forming a basis for pathogen discovery.

### 3.2 Distinct viral taxonomic patterns emerge in diseased ducklings across all levels of classification

**Figure 2:**
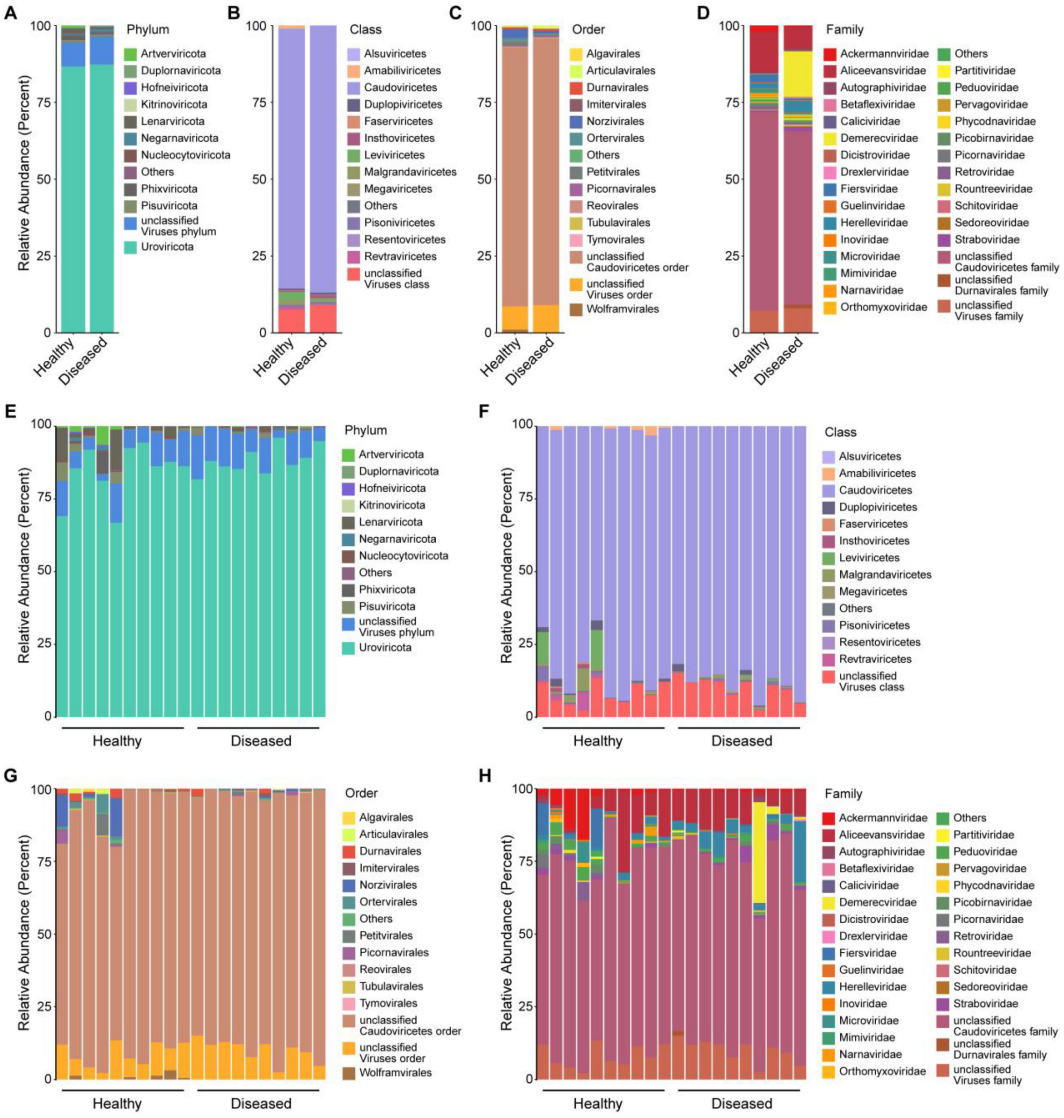
Taxonomic profiling of the duck intestinal virome. **(A–D)** Bar charts showing relative taxonomic abundances at the phylum to family levels, comparing healthy and diseased groups; **(E–H)** Sample-wise relative abundance plots at the phylum to family levels.

To further explore virome alterations, we annotated viral taxa from phylum to family, covering both bacteriophages and eukaryotic viruses. At the phylum level, *Uroviricota* dominated both groups but was substantially more abundant in diseased ducks (63.5%) compared to healthy ones (49.9%). *Negarnaviricota* and *Pisuviricota* were also more enriched in diseased ducklings, while *Phixviricota* appeared more prevalent in healthy individuals (**Figures 2A, E**). At the class level, *Caudoviricetes* was the most enriched taxon in diseased samples (63.5% vs. 49.9%). Additional enriched classes in the diseased group included *Leviviricetes*, *Insthoviricetes*, and *Malgrandaviricetes*, whereas *Amabiliviricetes* and *Revtraviricetes* were more common in the healthy group (**Figures 2B, F**). At the order level, unclassified Caudoviricetes were dominant in both groups, aligning with class-level patterns. Diseased ducks showed increased representation of *Articulavirales* and *Norzivirales*, while *Petitvirales* and *Ortervirales* were relatively more abundant in healthy individuals (**Figures 2C, G**). Family-level comparisons further highlighted marked differences in viral composition. *Demerecviridae* (12.3%), *Aliceevansviridae* (5.9%), and *Herelleviridae* (3.2%) were enriched in diseased ducks, whereas *Aliceevansviridae* (8.1%), *Fiersviridae* (1.6%), and *Ackermannviridae* (1.3%) were more abundant in healthy ducks. Notably, unclassified *Caudoviricetes* families were 7.5% more abundant in the diseased group (**Figures 2D, H**). A scatter plot depicting the relative abundances of the top 30 viral families across groups further corroborates these differential patterns, underscoring the distinct family-level viral profiles that distinguish healthy from diseased ducklings (**Supplementary Figure S1**).

### 3.3 Bacteriophage composition reveals gut virome shifts in diseased ducks

To gain deeper insight into virome structure and uncover potential pathogenic phages, we classified viruses based on host specificity—bacteriophages versus eukaryotic viruses—and performed family, genus, and species-level annotations of bacteriophages.

At the family level, *Demerecviridae*, *Straboviridae*, and *Autographiviridae* showed marked enrichment in diseased ducks (log_2_ FC > 3), while *Microviridae* and *Ackermannviridae* were relatively more abundant in healthy individuals (**Supplementary Figure S2**, **Supplementary Table S2**).

**Figure 3:**
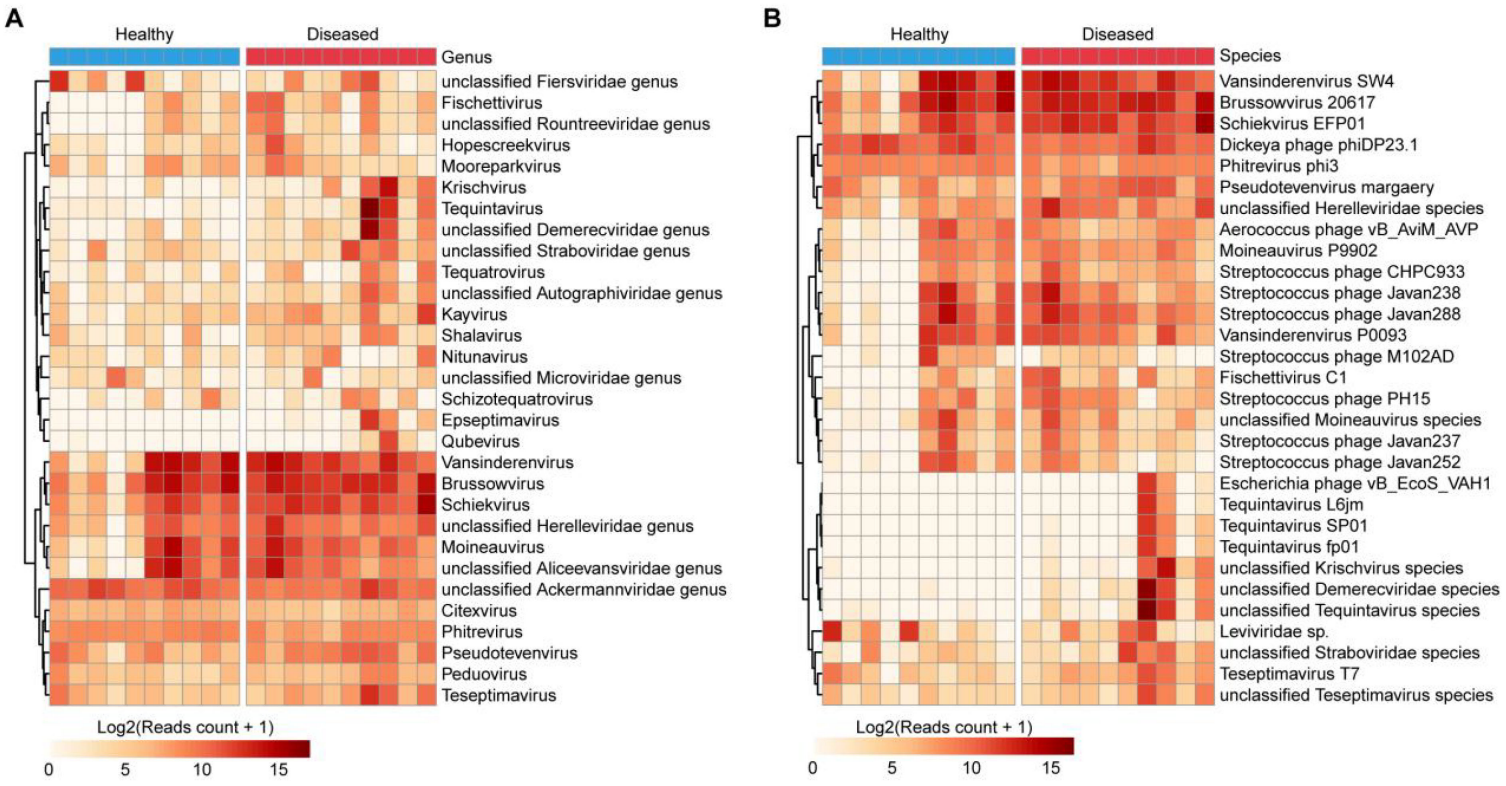
Characterization of gut-associated bacteriophages in ducks. **(A, B)** Heatmaps displaying the top 30 most abundant bacteriophage taxa at the genus **(A)** and species **(B)** levels across all samples.

At the genus level, *Brussowvirus*, *Vansinderenvirus*, *Tequintavirus*, *Schiekvirus*, and *Moineauvirus* represented dominant taxa in the duck gut. While *Brussowvirus*, *Vansinderenvirus*, and *Moineauvirus* were present at relatively low abundance in some healthy samples, no significant overall differences were observed between groups. However, *Tequintavirus* and *Schiekvirus* were markedly elevated in the diseased group, with approximate fold increases of 9,024 (log_2_ FC = 13.1) and 4.3 (log_2_ FC = 2.1), respectively, compared to the healthy group (**Figure 3A**, **Supplementary Table S3**). Several unclassified genera, such as unclassified *Aliceevansviridae*, unclassified *Demerecviridae*, unclassified *Ackermannviridae*, and unclassified *Herelleviridae*, were also prevalent in both groups. Notably, unclassified *Demerecviridae* showed a significant enrichment in the diseased group (483-fold; log_2_ FC = 8.9), whereas unclassified *Fiersviridae* was more abundant in the healthy group (2.8-fold; log_2_ FC = 1.5) (**Figure 3A**, **Supplementary Table S3**).

At the species level, the most abundant phages included *Brussowvirus* 20617, *Vansinderenvirus* SW4, unclassified *Tequintavirus* species, *Schiekvirus* EFP01, and unclassified *Demerecviridae* species, representing the core bacteriophage constituents of the Muscovy duck gut virome (**Figure 3B**, **Supplementary Table S4**). *Schiekvirus* EFP01 was significantly more abundant in diseased ducks (4.3-fold; log_2_ FC = 2.1), while *Pseudotevenvirus* margaery also showed elevated abundance in the diseased group (2.8-fold; log_2_ FC = 1.5). In contrast, *Leviviridae* sp. was depleted in diseased ducks, being 2.8-fold more abundant (log_2_ FC = 1.5) in healthy individuals (**Figure 3B**, **Supplementary Table S4**).

### 3.4 Differentially abundant phages highlight microbial shifts in disease

**Figure 4:**
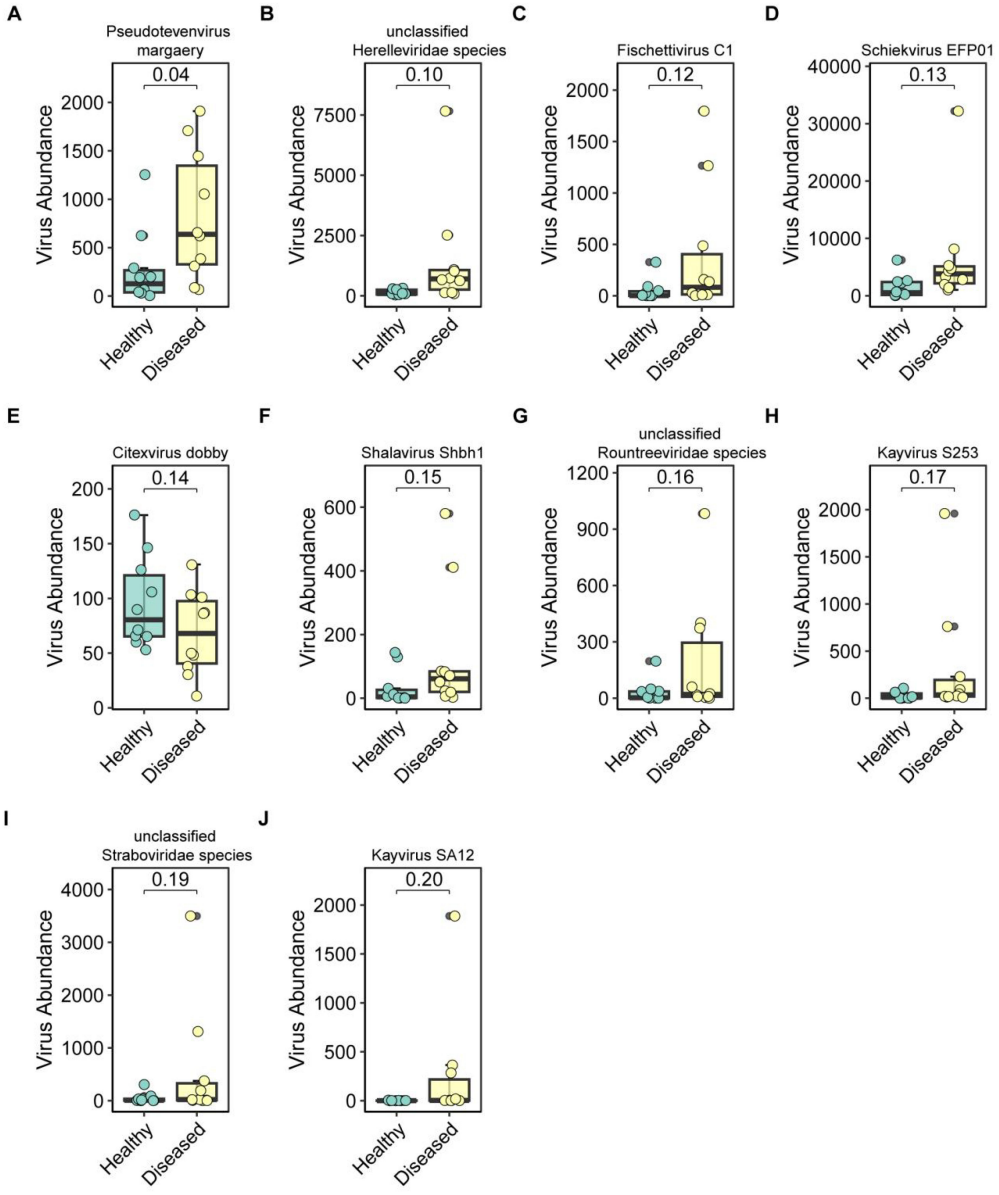
Differential abundance analysis of bacteriophages. **(A–J)** Top 10 bacteriophage taxa showing the most significant differences in abundance between healthy and diseased ducks.

To identify differentially abundant phage species, we performed a statistical comparison of relative abundances between groups and highlighted the top 10 significantly altered bacteriophages (**Figure 4**). These included *Pseudotevenvirus* margaery (p = 0.04; **Figure 4A**), unclassified *Herelleviridae* species (p = 0.10; **Figure 4B**), *Fischettivirus* C1 (p = 0.12; **Figure 4C**), *Schiekvirus* EFP01 (p = 0.13; **Figure 4D**), *Citexvirus* dobby (p = 0.14; **Figure 4E**), *Shalavirus* Shbh1 (p = 0.15; **Figure 4F**), unclassified *Rountreeviridae* species (p = 0.16; **Figure 4G**), *Kayvirus* S253 (p = 0.17; **Figure 4H**), unclassified *Straboviridae* species (p = 0.19; **Figure 4I**), and *Kayvirus* SA12 (p = 0.20; **Figure 4J**). These data indicate substantial shifts in phage community composition between healthy and diseased ducks, suggesting a possible role of phage dynamics in disease progression or microbiome imbalance.

### 3.5 Eukaryotic viral community structure varies between healthy and diseased ducks

To investigate the eukaryotic vertebrate-infecting viral community, we performed annotations at both genus and species levels. A heatmap of eukaryotic viral families highlights key abundance shifts: *Sedoreoviridae* and *Orthomyxoviridae* were notably enriched in diseased ducks (log_2_ FC > 1.9), while *Picobirnaviridae* and *Retroviridae* showed higher representation in healthy individuals(**Supplementary Figure S3**, **Supplementary Table S5**).

**Figure 5:**
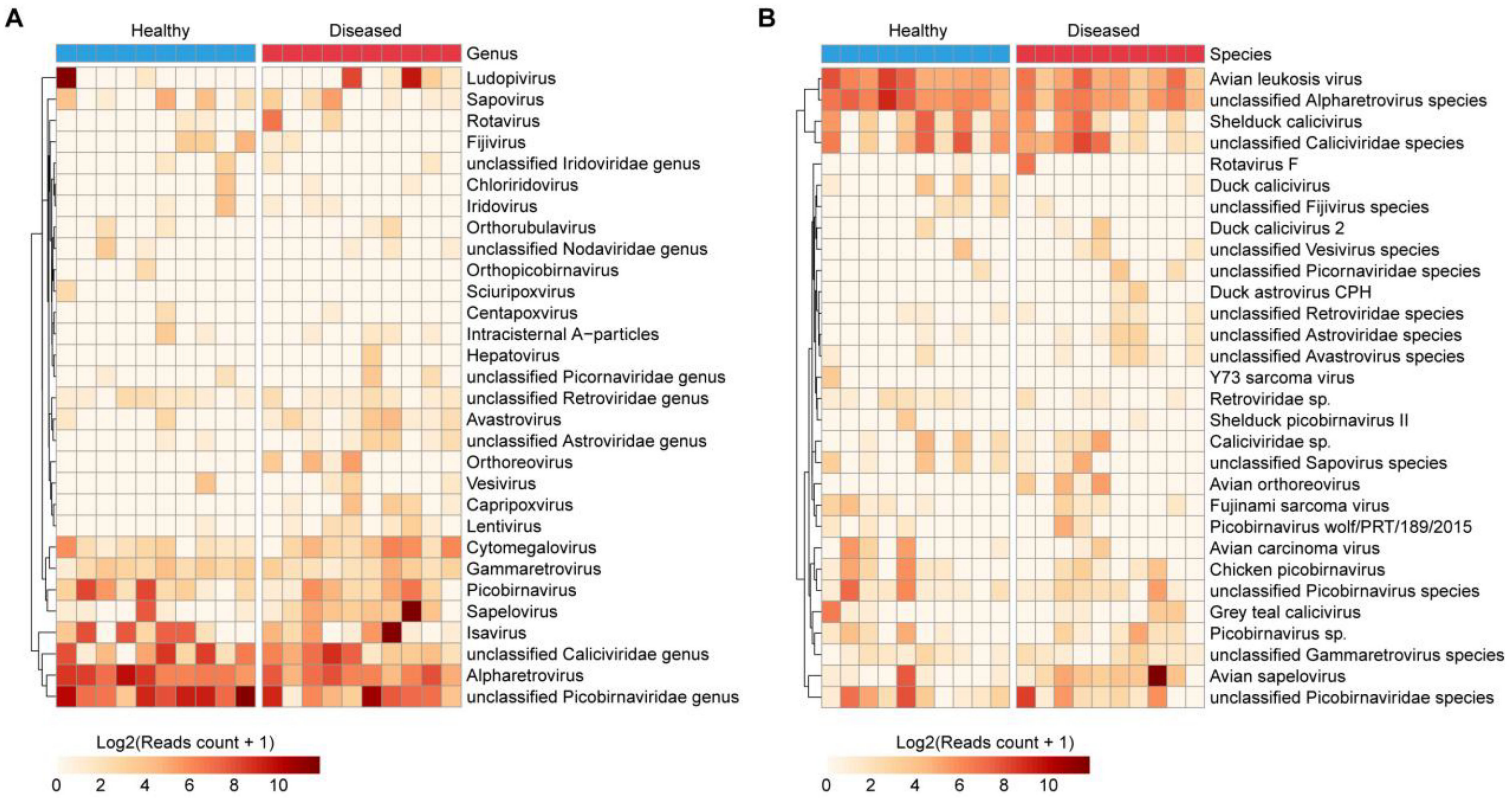
Composition of eukaryotic viruses in the duck gut. **(A, B)** Heatmaps illustrating the 30 most abundant eukaryotic viral taxa at the genus **(A)** and species **(B)** levels among all samples.

At the genus level, dominant taxa included unclassified *Picobirnaviridae*, *Ludopivirus*, *Sapelovirus*, unclassified *Caliciviridae*, and *Picobirnavirus*. Among these, *Sapelovirus* was significantly enriched in diseased ducks (18.4-fold; log_2_ FC = 4.2), while unclassified *Picobirnaviridae*, *Ludopivirus*, and *Picobirnavirus* were more prevalent in the healthy group (**Figure 5A**, **Supplementary Table S6**).

At the species level, the most abundant annotated viruses included *Avian sapelovirus*, unclassified *Caliciviridae* species, unclassified *Picobirnaviridae* species, *Shelduck calicivirus*, unclassified *Picobirnavirus* species, *Chicken picobirnavirus*, and *Grey teal calicivirus* (**Figure 5B**, **Supplementary Table S7**). Notably, *Avian sapelovirus* was significantly more abundant in diseased ducks (18.4-fold; log_2_ FC = 4.2). Furthermore, several viruses were detected exclusively in the diseased group but not in healthy individuals, such as *Avian orthoreovirus* and *Duck astrovirus* CPH (**Figure 5B**, **Supplementary Table S7**).

### 3.6 Differential abundance analyses reveal eukaryotic virus associations with disease

**Figure 6:**
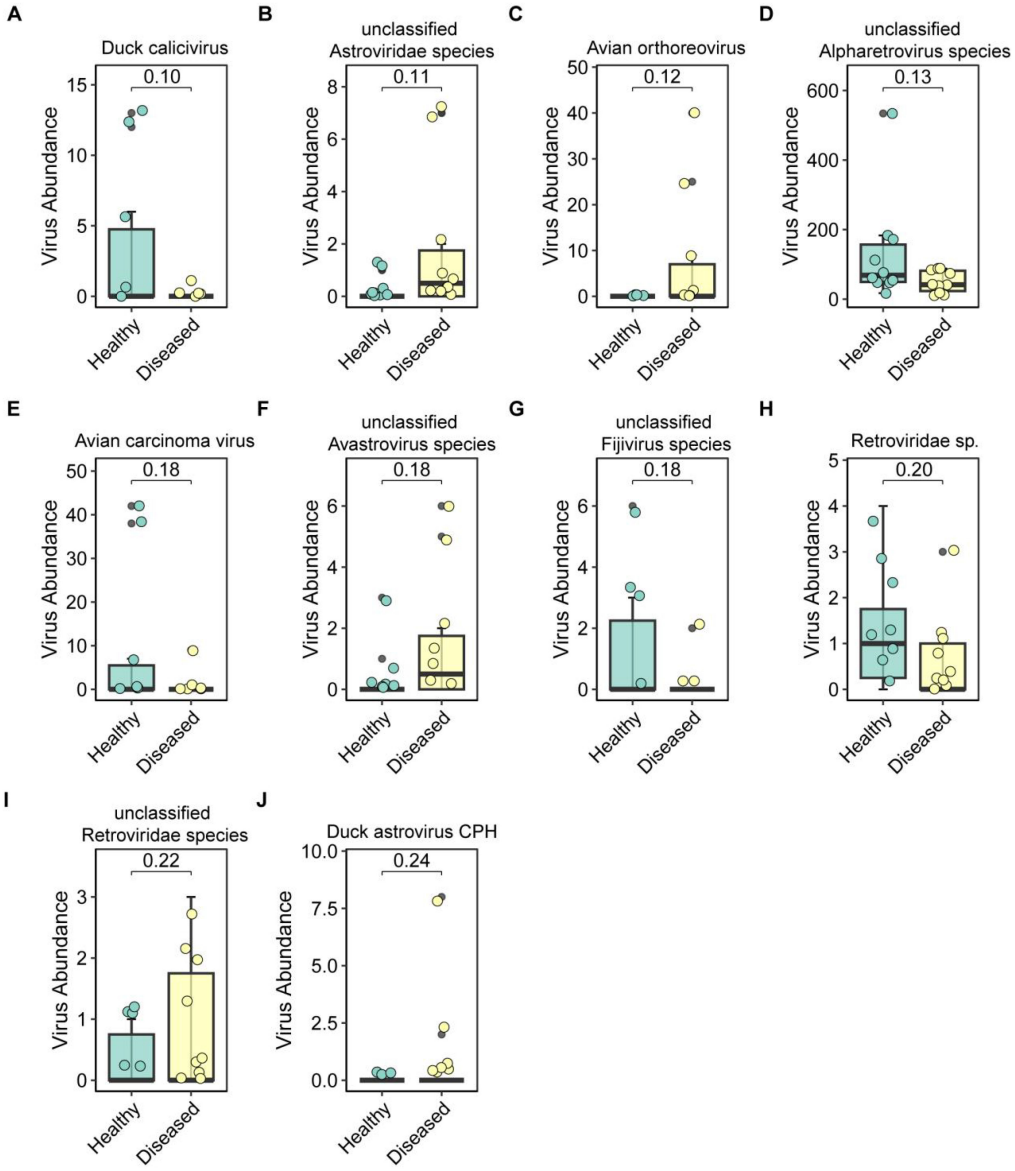
Differentially enriched eukaryotic viruses. **(A–J)** Top 10 eukaryotic viral taxa exhibiting the most significant abundance changes between healthy and diseased groups.

To identify significantly different eukaryotic viral species, differential abundance analysis was conducted, highlighting the top 10 differentially enriched viruses (**Figure 5**). These included *Duck calicivirus* (p = 0.10; **Figure 6A**), unclassified *Astroviridae* species (p = 0.10; **Figure 6B**), *Avian orthoreovirus* (p = 0.12; **Figure 6C**), unclassified *Alpharetrovirus* species (p = 0.13; **Figure 6D**), *Avian carcinoma virus* (p = 0.18; **Figure 6E**), unclassified *Avastrovirus* species (p = 0.18; **Figure 6F**), unclassified *Fijivirus* species (p = 0.18; **Figure 6G**), *Retroviridae* sp. (p = 0.20; **Figure 6H**), unclassified *Retroviridae* species (p = 0.22; **Figure 6I**), and *Duck astrovirus* CPH (p = 0.24; **Figure 6J**). These results further emphasize the distinct viral signatures associated with diseased ducks.

### 3.7 *Novel duck reovirus* is the primary candidate causative agent of the outbreak

**Figure 7:**
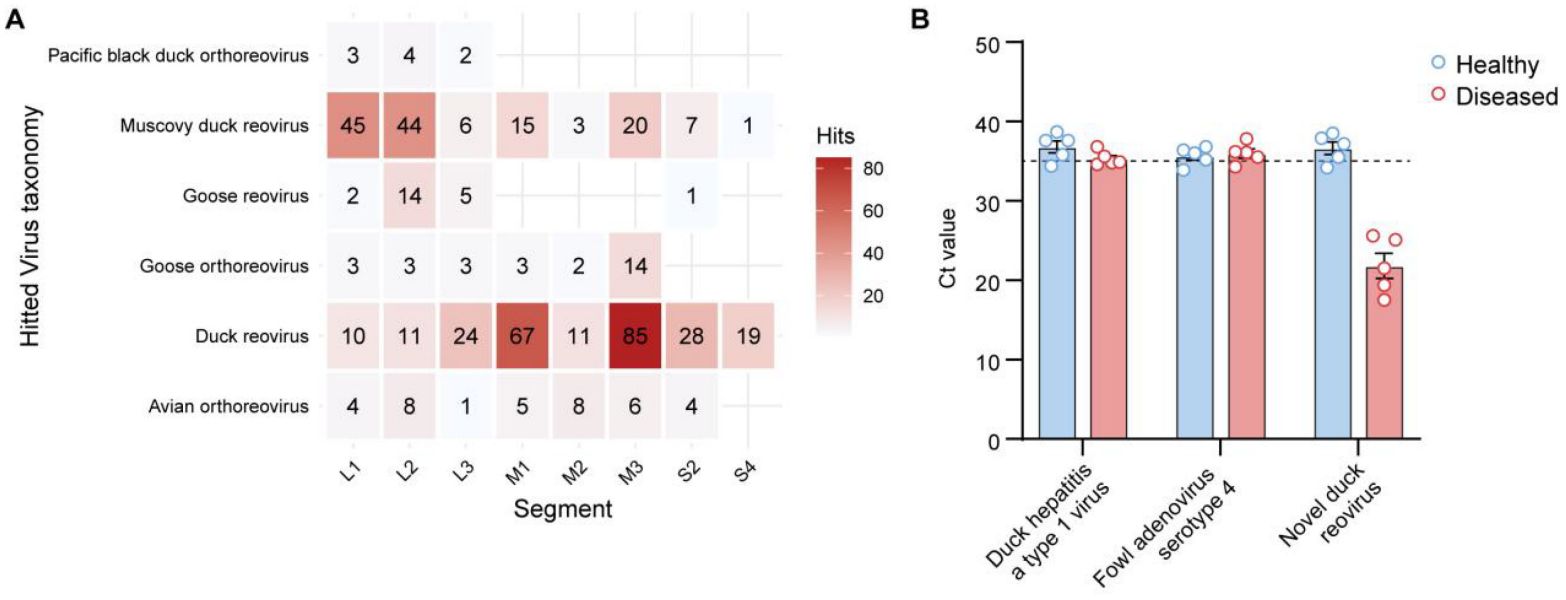
*Avian reovirus* identification and validation. **(A)** Taxonomic and gene-level annotations of *Avian reovirus* detected via metatranscriptomic sequencing; **(B)** Quantitative RT-PCR results confirming the presence of *Avian reovirus*.

Importantly, several viruses, including *Avian orthoreovirus*, were exclusively detected in the diseased group (**Figures 5B**, **6C**). Further analysis of *Avian orthoreovirus* sequences revealed annotations corresponding primarily to *Duck reovirus*, *Muscovy duck reovirus*, and *Goose orthoreovirus*. At the genomic level, annotated fragments included M3, M1, L1, and L2 segments (**Figure 7A**). Among these, *Novel duck reovirus* (NDRV)—a subtype of *Avian orthoreovirus*—has previously been associated with splenic and hepatic hemorrhages and necrosis in ducks (30). Based on our findings, we hypothesize that NDRV is the causative pathogen of the observed outbreak.

To validate this hypothesis, we performed RT-qPCR using primers specific for *Novel duck reovirus*, *Duck hepatitis A virus* (DHAV) and *Fowl adenovirus serotype 4* (FAdV-4). All diseased samples tested positive for *Novel duck reovirus*, while the other two viruses were not detected (**Figure 7B**). These findings provide strong support that *Novel duck reovirus* is the probable cause of the observed outbreak.

## 4 Discussion

In this study, we employed a metatranscriptomic approach for the first time to investigate the gut virome of healthy and diseased Muscovy ducks (*Cairina moschata*). To our knowledge, this is the first metatranscriptomic study of the Muscovy duck gut virome. Through differential analysis and RT-qPCR validation, we identified *Avian orthoreovirus* as a potential etiological agent responsible for hemorrhagic necrosis in the liver and spleen of diseased ducklings. This strategy presents a promising approach for rapid pathogen detection and the discovery of unknown infectious agents in poultry.

Accurate and timely diagnosis is essential for disease prevention and control not only in Muscovy duck farming but also across the broader livestock, wildlife industries and public health (31–33). This is particularly important when the potential etiological candidates are unknown. Advanced technologies such as high-throughput sequencing (HTS) have become indispensable tools for identifying potential pathogens under such circumstances, as demonstrated in uncovering the causes of diseases like Human Febrile Illness and Chapare Hemorrhagic Fever (34, 35). HTS has transformed molecular diagnostics, enabling comprehensive, scalable, and precise identification of pathogens within a short timeframe (36, 37). For instance, a nationwide study involving 1,941 mammalian samples from China identified 102 virus species from 13 families capable of infecting mammals, significantly broadening our understanding of wildlife viral diversity and strengthening surveillance strategies (19). In the context of livestock and poultry farming, HTS has already been applied in pathogen detection, diagnosis, and zoonotic disease surveillance. It has demonstrated significant advantages in identifying both known and emerging infectious agents, thereby enhancing the early warning capacity against animal-derived threats (38). While previous virome studies have explored the viral composition in passerine birds using metaviromics (39, 40), they have primarily focused on wild birds. Our investigation represents the first application of metatranscriptomics to characterize the gut virome of Muscovy ducks, offering a powerful diagnostic tool that can improve the efficiency, accuracy, and responsiveness of poultry disease management. Moreover, the adoption of such techniques contributes to the sustainable development of the farming industry and bolsters efforts to prevent zoonotic transmission.

Ducks, particularly wild waterfowl such as mallards (Anas platyrhynchos), are widely recognized as natural reservoirs or intermediate hosts for a variety of pathogens, including *influenza viruses*, *coronaviruses*, and *Escherichia coli* (41–43). These viruses circulate among wild waterfowl and frequently spill over into domestic poultry populations (44). In addition to *influenza viruses*, ducks can also carry other significant avian pathogens such as *Newcastle disease virus* (NDV) (45). Ducks, as natural hosts for multiple viruses, play a critical role in cross-species transmission, which holds significant public health implications (46). For instance, both gamma- and delta-coronaviruses have been detected in waterfowl, and ducks appear to be asymptomatic carriers. Ducks also contributed to the interspecies transmission of delta-coronaviruses from birds to pigs (47). In our study, only minimal reads were assigned to *coronaviruses* and *influenza viruses*, suggesting that Muscovy ducks were unlikely infected with these pathogens. This observation may also reflect the success of current vaccination and biosecurity measures implemented in Guangdong’s duck farming systems. Therefore, characterizing the virome of ducks not only has implications for their health management but also plays a vital role in public health. In-depth virome studies in ducks could inform effective disease control strategies and help anticipate and prevent cross-species transmission events.

Previous metatranscriptomic analyses of duck fecal viromes have identified a range of viral families, including *Paramyxoviridae*, *Narnaviridae*, *Bunyaviridae*, *Birnaviridae*, and *Reoviridae* (48). Viral metatranscriptomics has also enabled the discovery of novel viruses in species like ruddy shelducks, underscoring the importance of continuous monitoring for emerging threats to both wildlife and public health (49). Some viruses once thought to be restricted to Southeast Asia have since been detected in northern Eurasia, indicating wider geographic distribution than previously assumed (50). These findings reinforce the role of ducks as viral reservoirs and potential conduits for pathogen persistence and transmission. Our study revealed significant restructuring of the bacteriophage community in diseased ducks (**Figures 3**, **4**). Notably, lytic bacteriophage families typically associated with pathogenic bacteria (*Demerecviridae* and *Straboviridae*) were markedly enriched in the diseased group, whereas temperate phage taxa implicated in microbiome homeostasis maintenance (*Microviridae* and *Ackermannviridae*) showed relative attenuation. This bidirectional imbalance pattern—characterized by lytic phage proliferation alongside temperate phage suppression—may synergize with intestinal microbial dysbiosis in diseased ducks (51, 52), ultimately contributing to microecological collapse. Future work should investigate the contributions of these viral groups to disease processes in Muscovy ducks. In addition to novel virus discovery, virome sequencing provides insights into viral evolution, recombination, and transmission patterns (40). Additionally, Genome assembly can uncover recombination events that may generate new viral strains, potentially impacting vaccine efficacy and posing economic risks to poultry production (53). Therefore, a comprehensive and integrative approach to studying the Muscovy duck virome is vital for understanding the interplay between viruses, hosts, and the environment, ultimately promoting sustainable poultry development.

*Novel duck reoviruses* (NDRV) have been associated with more severe clinical presentations than classical *Muscovy duck reoviruses*, particularly extensive hemorrhagic necrosis in the spleen and liver, highlighting their high pathogenic potential (54). NDRV infection has also been linked to immunosuppression, increasing susceptibility to secondary bacterial or viral infections (55). In our study, we observed marked hemorrhagic necrosis in the liver and spleen (**Figure 1A**). Metatranscriptomic analysis revealed a clear increase in *Avian orthoreovirus* abundance in diseased ducks, whereas no such signal was detected in healthy individuals. Avian orthoreoviruses in ducks primarily include Muscovy duck reovirus (MDRV) and NDRV. MDRV typically manifests with disseminated white necrotic foci in the liver and intestinal mucosal damage (56, 57), whereas NDRV is characterized by splenic necrosis, swelling, and hepatic lesions (58). Integrating clinical symptoms with multi-omics detection results, we conclude that these pathological changes were likely caused by NDRV, a conclusion further supported by RT-qPCR validation. While NDRV is a strong candidate, causal confirmation requires future isolation and challenge studies. It should be noted that the universal NDRV positivity in diseased samples by RT-qPCR appears discordant with the relatively low abundance of Avian orthoreovirus in some samples (**Figure 6C**). This apparent discrepancy is closely associated with technical limitations in sequencing depth. Our selection of moderate depth was driven by practical application considerations for future field deployment—excessive depth increase would substantially raise per-sample costs, hindering large-scale implementation. Crucially, the complete absence of NDRV detection in healthy ducks (**Figures 5B**, **6C**) confirms assay specificity. While our study provides new insights into the diagnosis and identification of viral pathogens in Muscovy ducks, further direct evidence, such as virus isolation and animal challenge experiments, is necessary to definitively confirm the pathogenic role of NDRV. Although metatranscriptomic genome assembly and evolutionary analysis are valuable tools, the relatively low abundance of reovirus sequences in this study precluded full genome assembly or phylogenetic characterization. Future research will focus on improving sampling strategies, sequencing depth, and assembly algorithms, potentially incorporating artificial intelligence tools (12) maximize the diagnostic power of metatranscriptomics in uncovering novel pathogens.

## Data Availability

The datasets presented in this study can be found in online repositories. The names of the repository/repositories and accession number(s) can be found in the article/**Supplementary Material**. The sequencing data that support the findings of this study has been deposited in the National Center of Biotechnology Information (NCBI) Sequence Read Archive (SRA) and is accessible via the accession number SRR34855210–SRR34855229.

## Supplementary material

Supplementary Figure 1 Scatter plot showing the relative abundances of the top 30 viral families across different experimental groups.

Supplementary Figure 2 Heatmap presenting sample-wise total abundance rankings for the top 30 phage families at the family level.

Supplementary Figure 3 Heatmap depicting the total abundance rankings of the top 30 eukaryotic viral families in each sample at the family level.
