# Supplementary material for: Metatranscriptomic analysis of the gut virome in Muscovy ducks reveals a novel duck reovirus potentially associated with hepatic and splenic hemorrhage
Source: Front Immunol. 2025 Oct 23;16:1680275. doi: 10.3389/fimmu.2025.1680275 (PMC12588956; doi:10.3389/fimmu.2025.1680275)
Supplement: Supplementary Figure 1 — Scatter plot showing the relative abundances of the top 30 viral families across different experimental groups. [file DataSheet2.docx]

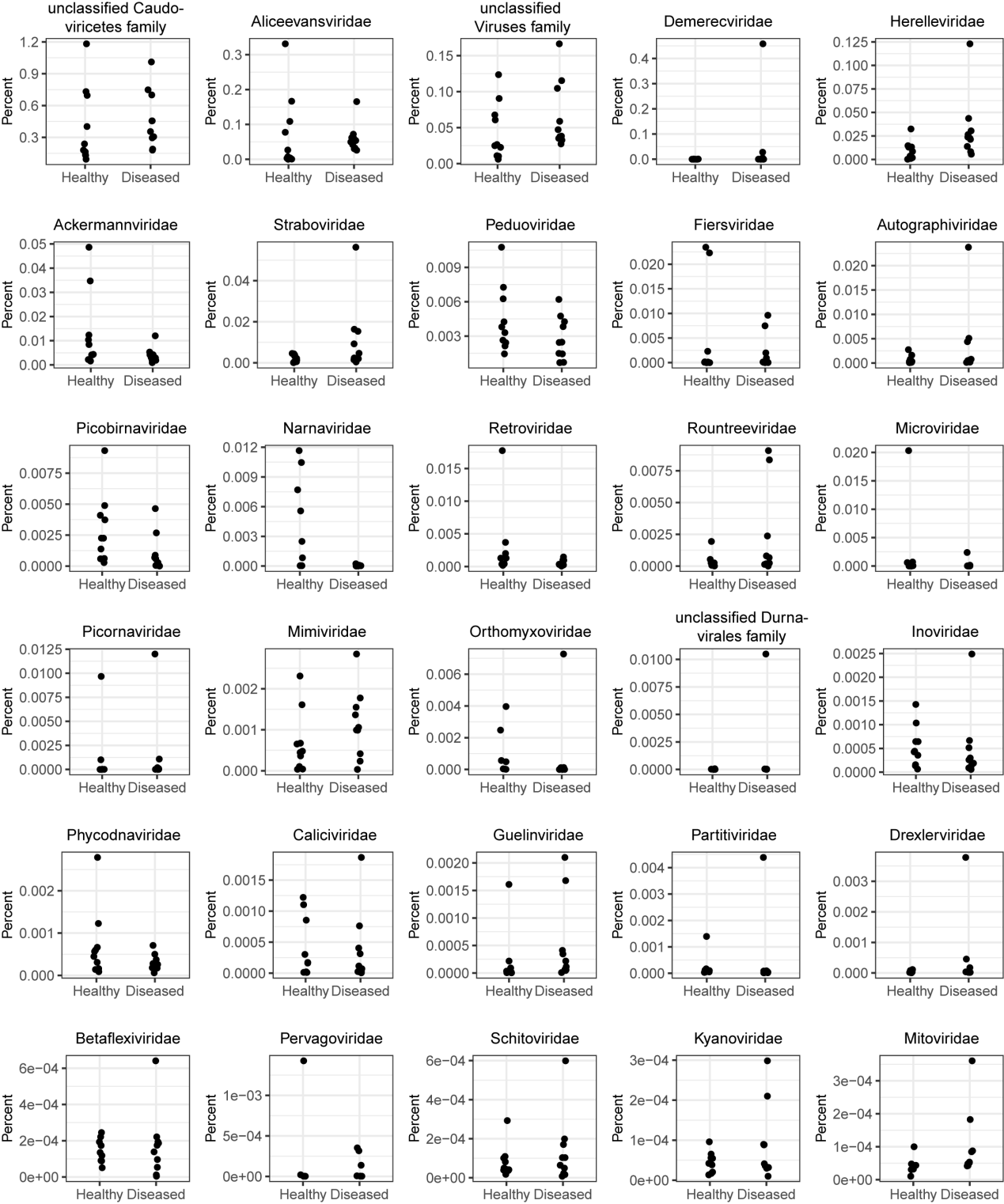


Supplementary Figure 1. Scatter plot showing the relative abundances of the top 30 viral families across different experimental groups.


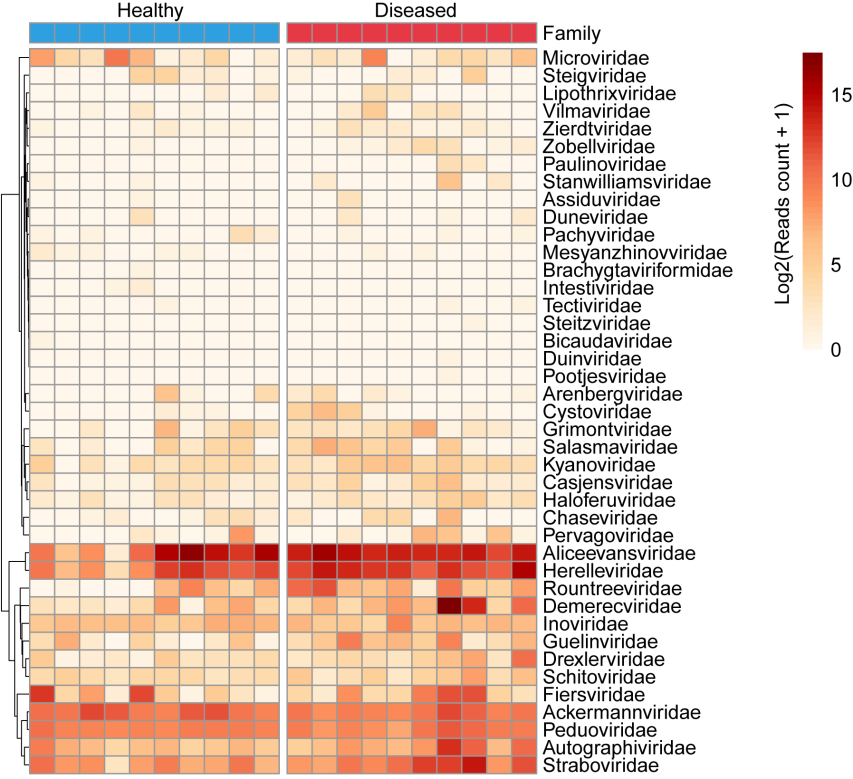


Supplementary Figure 2. Heatmap presenting sample-wise total abundance rankings for the top 30 phage families at the family level.


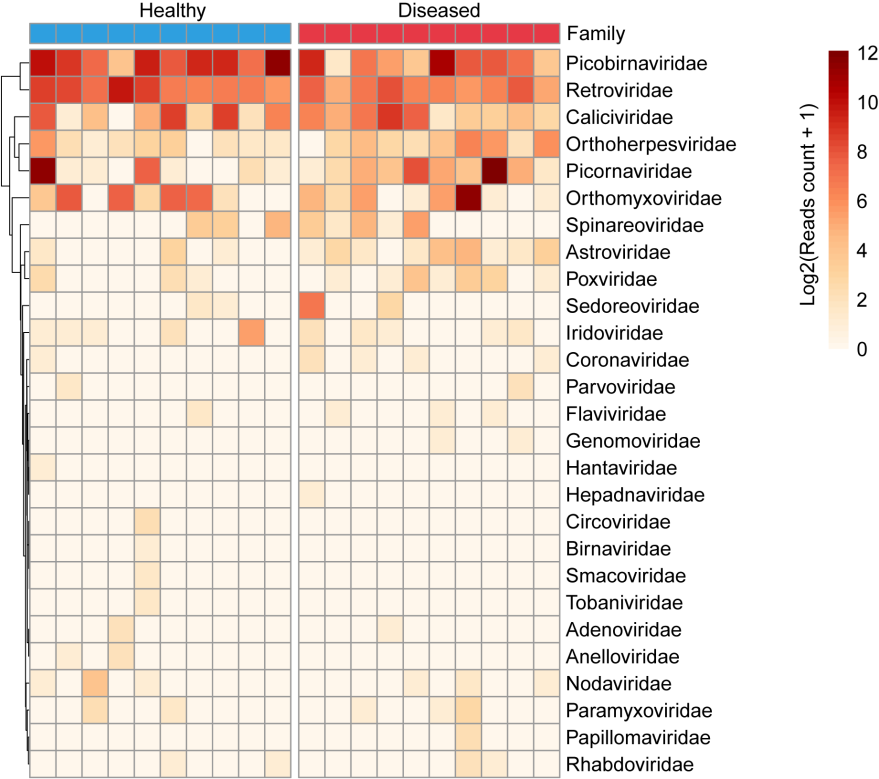


Supplementary Figure 3. Heatmap depicting the total abundance rankings of the top 30 eukaryotic viral families in each sample at the family level.
